# A Motion-Activated Video Game for Prevention of Substance Use Disorder Relapse in Youth: Pilot Randomized Controlled Trial

**DOI:** 10.2196/11716

**Published:** 2019-05-23

**Authors:** Lorien C Abroms, Marc Fishman, Hoa Vo, Shawn C Chiang, Victoria Somerville, Lawrence Rakhmanov, Michael Ruggiero, Daniel Greenberg

**Affiliations:** 1 Department of Prevention and Community Health Milken Institute School of Public Health The George Washington University Washington, DC United States; 2 Mountain Manor Treatment Center Baltimore, MD United States; 3 Media Rez LLC Washington, DC United States

**Keywords:** youth, addiction treatment, opioid, marijuana, video game, technology

## Abstract

**Background:**

Body motion-activated video games are a promising strategy for promoting engagement in and adherence to addiction treatment among youth.

**Objective:**

This pilot randomized trial (N=80) investigated the feasibility of a body motion–activated video game prototype, Recovery Warrior 2.0, targeting relapse prevention in the context of a community inpatient care program for youth.

**Methods:**

Participants aged 15-25 years were recruited from an inpatient drug treatment program and randomized to receive treatment as usual (control) or game play with treatment as usual (intervention). Assessments were conducted at baseline, prior to discharge, and at 4 and 8 weeks postdischarge.

**Results:**

The provision of the game play intervention was found to be feasible in the inpatient setting. On an average, participants in the intervention group played for 36.6 minutes and on 3.6 different days. Participants in the intervention group mostly agreed that they would use the refusal skills taught by the game. Participants in the intervention group reported attending more outpatient counseling sessions than those in the control group (10.8 versus 4.8), but the difference was not significant (*P*=.32). The game had no effect on drug use at 4 or 8 weeks postdischarge, with the exception of a benefit reported at the 4-week follow-up among participants receiving treatment for marijuana addiction (*P=*.04).

**Conclusions:**

Preliminary evidence indicates that a motion-activated video game for addiction recovery appears to be feasible and acceptable for youth within the context of inpatient treatment, but not outpatient treatment. With further development, such games hold promise as a tool for the treatment of youth substance use disorder.

**Trial Registration:**

ClinicalTrials.gov NCT03957798; https://clinicaltrials.gov/show/NCT03957798 (Archived by WebCite at http://www.webcitation.org/78XU6ENB4)

## Introduction

Drug use is recognized as a serious public health problem among adolescents and young adults. In 2015, 37.5% of young adults aged 18-25 years and 17.5% of adolescents aged 12-17 years in the United States reported the use of illicit drugs in that year [1]. Adolescent and young adult substance use disorders (SUDs) are associated with numerous negative outcomes including overdose, HIV transmission, school failure, criminal behavior, and other social problems.

The standard of care for youth with SUDs includes detoxification as needed, followed by traditional psychosocial treatments [2-4]. Psychosocial treatments typically consist of individual and group counseling and may focus on developing skills related to abstinence, such as problem solving, coping, and refusal skills [5]. Although such programs are associated with positive outcomes for youth [5], dropout from treatment remains a major barrier to success [6]. There is a need to develop innovative strategies to improve retention among youth and increase the rates of abstinence.

One promising strategy to promote treatment engagement and adherence is to create models of treatment that offer therapeutic content in game-based formats. Games, including video games, have been explored as therapeutic tools for alleviating a variety of psychological and physical conditions such as stress, anxiety, and mood disorders [7] as well as for treating addiction [8-12]. For addiction, video games have been used to change knowledge and risk perception surrounding drugs and alcohol, develop refusal skills, and help people quit smoking [10-13]. Such games have involved role play [12] and virtual reality exploration [13]. However, it is unclear how experiential games such as motion-activated games using platforms such as the Nintendo Wii and Microsoft Kinect can be used in addiction treatment.

This study builds on an earlier pilot study [14] and examines how a game that runs on an off-the-shelf gaming system (Microsoft Kinect) can be used in SUD treatment by helping patients develop negative associations with drugs and acquire drug-refusal skills [15]. This study is a pilot randomized trial (N=80) of a revised body motion–activated game, Recovery Warrior 2.0, targeting relapse prevention in the context of a community treatment program for SUD among youth. Of interest was the feasibility of the game in the inpatient and outpatient settings, participant ratings of the game, the effect of the game on the mediators of relapse, treatment adherence and retention, and drug use outcomes.

## Methods

### Study Procedures

The study was approved by the MaGil Institutional Review Board. Participants were recruited from the short-term inpatient program at the Mountain Manor Treatment Center (MMTC) in Baltimore, MD, between February 5, 2016, and June 21, 2016.

Patients were approached by MMTC research staff about participating in the study within their first few days of inpatient admission, allowing some time for adjustment to the environment and resolution of the most acute phase of withdrawal distress. Interested individuals were assessed for eligibility and, if eligible, provided written consent. For patients under the age of 18 years, assent and parental consent were obtained.

Inclusion criteria were as follows: age of 15-25 years, attending the MMTC inpatient program for primarily opioid or marijuana use disorder treatment, ability to speak English, absence of a comorbid psychiatric condition that would make participation unsafe (eg, acute suicidality or unstable psychosis), and no pregnancy (because of the physical exertion required to play the game).

**Figure 1:**
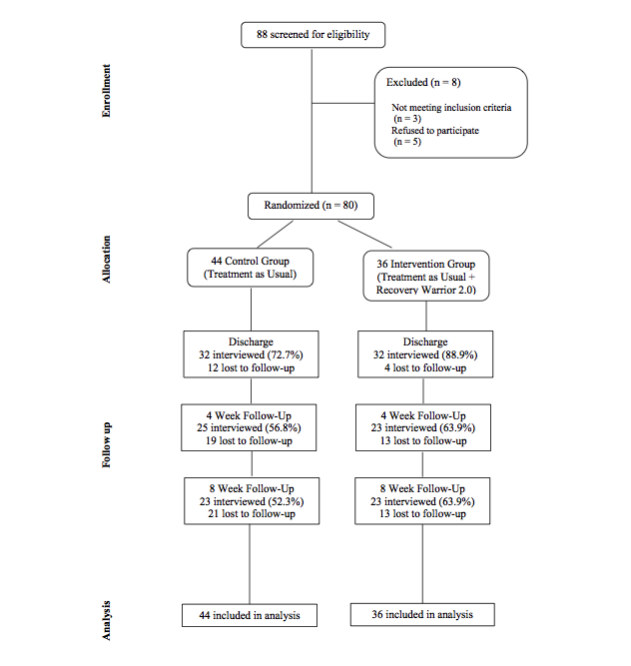
CONSORT flow diagram. Participant enrollment and follow-up.

Once consent was obtained, the participants were given a baseline survey and randomized to receive Recovery Warrior game play with treatment as usual or to receive treatment alone. In addition to the baseline survey, all participants were given an in-person survey prior to inpatient discharge (discharge survey) and another survey by phone at 4 weeks and 8 weeks after discharge from inpatient treatment. Participants were given a US $20 gift card for each survey, plus a bonus gift card of US $10 at 4 weeks and US $20 at 8 weeks. This resulted in a maximum incentive of US $110 for assessments. Phone calls, text messages, Facebook messages, and subject interception at MMTC outpatient treatment were used to remind participants of their upcoming follow-up surveys. For the 4- and 8-week surveys, up to 15 contact attempts were made per survey before considering the case as a missed follow-up. Participant flow can be seen in Figure 1.

### Recovery Warrior Game Play With Treatment as Usual

In addition to their usual care, participants randomized to game play were given the opportunity to participate in a game play session 3 times/week for the length of their stay in the residential (inpatient) program. Typical inpatient stays at MMTC are for 9 days, and thus, it was expected that participants would have 4 game play sessions over the course of their inpatient stay. Participants in the intervention group who transitioned to outpatient care at MMTC were given an additional weekly opportunity to play the game for 4 weeks. The goal was for each game play session to last 1 hour and include 3-5 participants, with each participant playing for at least 10 minutes and no more than 15 minutes per session. Players would take turns, with each player playing one at a time and the others watching and encouraging him/her. Each 1-hour session included an introduction to the game by the counselor (2 min), game play, and an informal debriefing by the counselor about lessons learned in the game (8-10 mins). In the first session, the participants were directed to play each game, so that they would have an experience of each of the games. Subsequently, participants could choose to play any of the games. Sessions were offered in a dedicated room at the MMTC.

Recovery Warrior 2.0 [16] was developed for use with Microsoft Kinect running on a Windows personal computer. The 2.0 version was improved from an initial version that was previously pilot tested [17] and consisted of a suite of several games. All games made use of whole-body motion detection and the same voice-recognition feature. Body motions included a variety of arm, leg, and whole-body movements to physically enact the motions of destroying or evading images of drugs and drug paraphernalia. Voice features consisted of recognition of the refusal phrase “I’m Clean” Players could say or shout “I’m Clean” in order to gain additional strength for their game play avatar. All game art was created in a hyperrealistic, idealized, heroic style, which is the preferred style choice as per a focus group in an earlier work [17]. Across games, players were given a choice of several distinct hyperrealistic avatars. The counselor set up the game, so that the drug images would correspond to the drug being treated for (eg, opioid patients would see syringes, spoons, pill bottles, and pills as part of game play, while marijuana patients were exposed to marijuana cigarettes, baggies of marijuana, and bongs). Players could choose whether to play in a mode where they destroyed drugs, avoided drugs, or discerned prosocial “goodies” from drugs while avoiding drugs and collecting “goodies.” Goodies included images of items such as graduation caps, car keys, and footballs.

The following games were tested: Recovery Ninja (destroy drugs), Recovery Ninja+Goodies (destroy and discern), Recovery Climber (avoid drugs), Recovery Racer (destroy drugs), Recovery Racer+Goodies (destroy and discern), Recovery Runner (avoid drugs), and Recovery Runner+Goodies (avoid and discern). For example, the goal of Recovery Ninja is to destroy drugs that fly at the player’s avatar. The player must make chopping, punching, and hitting gestures to destroy the drugs that fly across the screen in order to win the game while periodically shouting “I’m Clean” to power up. Another example is Recovery Runner. In this game, the player runs through a dark city, which progressively brightens as the player succeeds in staying away from drugs. Instead of destroying drugs (as in Recovery Ninja), the player must avoid them by physically ducking, dodging, and jumping to control the avatar’s movements and avoid touching the drugs. As with other games, the player periodically shouts “I’m Clean” to gain additional power. See Multimedia Appendix 1 for more details and screenshots of the games.

### Theoretical Mechanism of Recovery Warrior

The development of the game was based on the social cognitive theory, repetition priming, and the reinforcement theory of motivation [17,18]. Developed first in 1960, the social cognitive theory considers ways in which individuals acquire and maintain new behaviors while considering the social environment in which the individuals perform the behavior. Based on social cognitive theory, it is hypothesized that by repeatedly role playing destroying drugs/avoiding drugs in the context of the game, players will experience increases in their self-efficacy and behavioral capability for drug refusal and avoidance in the real world [18]. This may occur because players develop self-schemas of themselves as drug destroyers or avoiders rather than users [19]. Furthermore, drug refusal skills and self-schemas as nonusers will be further enhanced by the constant repetition of the phrase “I’m Clean” throughout the game, so that participants will be primed to use it if offered drugs in a future situation [20].

Additionally, based on the reinforcement theory of motivation, we hypothesize that youth will be better able to learn these skills if the learning process is paired with rewards. In this case, rewards associated with playing immersive video games may include positive feelings as a sense of mastery; eustress; and pleasure, activation, and arousal from the game-based exercise and physical exertion [21,22]. Finally, because this game is being designed as a social game to be played in the company of others in treatment, it is also hypothesized that social learning will contribute to the mastery of refusal skills and drug avoidance [18]. Participants will learn the skills of avoiding drugs/refusing drugs by not only repeatedly playing themselves, but also watching others practice these skills in the context of the game.

### Treatment as Usual

Treatment as usual at the MMTC consisted of individual and group counseling as well as pharmacotherapy, where recommended. MMTC is a Joint Commission-accredited community treatment program for SUDs and co-occurring mental health conditions. Typically, patients stay in the inpatient program for 1-2 weeks and then transition to the outpatient program at the MMTC or another treatment center.

### Measures

The baseline survey included measures of the demographic characteristics of participants, video game use, and history of drug use of participants. Participants were asked about the primary drug that they were in treatment for. Opioid and marijuana use at follow-up was ascertained by self-report of any use in the past 7 and 30 days, using the Time Line Follow Back tool, as well as the date of last use.

For the intervention group at the 4-week follow-up, participants were asked about their perceptions of the most helpful game among the games played and the mode of game play that was seen as most helpful (eg, destroy drugs, avoid drugs, and collect goodies). Computer records of game play were also used to measure minutes of game play for each participant and days of game play. Measures of user engagement in Recovery Warrior were collected through a retrospective review of the computer records from game play. The system recorded each time a user played the game in minutes of game play. For each participant, the number of total minutes of game play was calculated across the intervention period.

Additionally, the 4-week follow-up survey assessed refusal skills taught by the game. Refusal skills were measured by asking participants if they agreed that they would use the phrase “I’m Clean” to refuse drugs (1=not agree to 5=highly agree), if they had used the phrase “I’m Clean” since discharge to refuse drugs, and if the phrase “I’m Clean” still rings in their head (not at all, less than once per week, a few times a week, or more often).

For both groups, psychosocial mediators of recovery, self-efficacy, and cravings were measured. Self-efficacy for refusal of drugs was measured using the Marijuana Resistance Self-Efficacy scale at baseline, discharge, and follow-up surveys [23,24]. It used a 4-item, 4-point scale (1=very easy to 4=very hard) that asked participants how easy or hard it would be to refuse the drug if offered and explain why they did not want it, why they wanted to avoid the situation in the first place, and why they wanted to leave the situation. It was adapted so that there was a similar version for opioid use. Participants were only asked about the primary drug for which they enrolled in treatment (ie, marijuana or opioids).

For cravings, the 5-item Penn Alcohol Craving Scale [25] was included at baseline, discharge, and postdischarge follow-up surveys, but modified to apply to marijuana and opioid use. It assessed the intensity of a participant’s cravings (0=none at all to 6=very strong; sum of a maximum total of 30 points).

Treatment rating was measured in three ways. First, it was measured with the Counselor Alliance Scale, which was taken from the Working Alliance Inventory [26-28], and used to measure treatment progress with the counselor at discharge, 4 weeks, and 8 weeks. The Counselor Alliance Scale uses 7-items and 7-points to measure how well participants believe counselors are working with them to improve their situation (1=never to 7=always). The treatment rating was also measured by asking participants about their satisfaction with inpatient care at the time of discharge and satisfaction with outpatient care at the 4-week and 8-week follow-up surveys.

Treatment use was measured by self-report of the use of outpatient services including meeting a doctor, meeting a counselor, attending group sessions, taking medications, and other services. The total number of services used was summed up for each participant. Participants were also asked at the 4-week and 8-week follow-ups about the number of outpatient counseling sessions attended in the past 30 days. Drug use outcomes were measured by asking participants at the 4-week and 8-week surveys if they had used the drug for which they received treatment (eg, opiates or marijuana), over the past 7 and 30 days.

### Analysis

Means and SDs or percentages were calculated for key variables and compared between intervention and control groups. Chi-squared tests were used for categorical variables, and two-tailed *t* tests were used for continuous variables. Outcome analyses were conducted both with collected data alone and with missing values imputed as positive for drug use. In addition to the combined analyses, outcome analyses were conducted separately for marijuana and opioid patients.

## Results

### Participant Characteristics

Eighty participants were recruited, of which 36 were randomized to the intervention group and 44 were randomized to the control group. Of the 80 participants, 64 completed the discharge interview (80.0%), 48 completed the 4-week follow-up interview (60.0%), and 46 completed the 8-week follow-up interview (57.5%). There were no significant differences between groups in terms of survey completion.

Most participants were between the ages of 18 and 20 years. More than half of the participants were not attending school at the time of the study (65.0%), while 26.3% were in high school and the other 8.8% were in college. The majority of participants were male (77.5%). Most participants identified themselves as white (63.8%) or black (28.8%). Half of the participants had a mother who finished high school/General Education Development as the highest level of education (50.0%) and almost half had a father with a similar level of education (46.3%). Participants were in treatment for opioid (57.5%) or marijuana (42.5%) use disorder. Almost half of the participants reported daily prestudy video game use (46.3%) and, to a lesser extent, weekly use (18.8%), monthly use (23.8%), and none at all (11.3%). Most participants in our sample spent 4-6 days at the MMTC (51.3%), and fewer spent 7-10 days (31.3%) (Table 1).

**Table 1:** Demographic characteristics and drug use history of participants.

Characteristics		All participants (N=80)	Intervention group (N=36)	Control group (N=44)
**Age (years), mean (SD)**				
	<18	15 (18.8)	9 (25.0)	6 (13.6)
	18-20	44 (55.0)	21 (58.3)	23 (52.3)
	21-25	21 (26.3)	6 (16.7)	15 (34.1)
**Grade, n (%)**				
	8th-12th	21 (26.3)	13 (36.1)	8 (18.2)
	College	7 (8.8)	1 (2.8)	6 (13.6)
	Not in school	52 (65.0)	22 (61.1)	30 (68.2)
**Gender, n (%)**				
	Male	62 (77.5)	32 (88.9)	30 (68.2)^a^
	Female	18 (22.5)	4 (11.1)	14 (31.8)
**Race/ethnicity, n (%)**				
	Black	23 (28.8)	9 (25.0)	14 (31.8)
	White	51 (63.8)	23 (63.9)	28 (63.6)
	Other^b^	6 (7.5)	4 (11.1)	2 (4.5)
**Mother’s education, n (%)**				
	Did not graduate high school	9 (11.3)	3 (8.3)	6 (13.6)
	High school graduate/GED^c^	40 (50.0)	20 (55.6)	20 (45.5)
	College or higher	27 (33.8)	10 (27.8)	17 (38.6)
	No response	4 (5.0)	3 (8.3)	1 (2.3)
**Father’s education, n (%)**				
	Did not graduate high school	14 (17.5)	7 (19.4)	7 (15.9)
	High school graduate/GED	37 (46.3)	13 (36.1)	24 (54.5)
	College or higher	12 (15.0)	7 (19.4)	5 (11.4)
	No response	17 (21.3)	9 (25.0)	8 (18.2)
**Primary drug of treatment, n (%)**				
	Marijuana	34 (42.5)	18 (50.0)	16 (36.4)
	Opiates	46 (57.5)	18 (50.0)	28 (63.6)
**Ever used intravenous drugs,** **n (%)**				
	Used	37 (46.3)	16 (44.4)	21 (47.7)
	Did not use	43 (53.8)	20 (55.6)	23 (52.3)
**Used any drug in the past 7 days, n (%)**				
	Yes	41 (51.3)	18 (50.0)	23 (52.3)
	No	39 (48.8)	18 (50.0)	21 (47.7)
**Date last drug used , n (%)**				
	<1 month	72 (90.0)	31 (86.1)	41 (93.2)
	1-2 months	8 (10.0)	5 (13.9)	3 (6.8)
	>2 months	0 (0.0)	0 (0.0)	0 (0.0)
**Current video game use, n (%)**				
	Most days/daily	37 (46.3)	14 (38.9)	23 (52.3)
	1-2 times/week	15 (18.8)	5 (13.9)	10 (22.7)
	Few times a month	19 (23.8)	12 (33.3)	7 (15.9)
	Not at all	9 (11.3)	5 (13.9)	4 (9.1)
**Total days at the Mountain Manor Treatment Center, n (%)**				
	1-3 days	10 (12.5)	4 (11.1)	6 (13.6)
	4-6 days	41 (51.3)	17 (47.2)	24 (54.5)
	7-10 days	25 (31.3)	12 (33.3)	13 (29.5)
	≥10 days	4 (5.0)	3 (8.3)	1 (2.3)
Baseline craving, mean (SD)		7.8 (3.5)	8.1 (3.2)	7.7 (3.8)
Baseline self-efficacy, mean (SD)		9.2 (3.6)	8.7 (3.5)	9.7 (3.8)

^a^*P*=.03.

^b^The category “other” includes Asian, Hispanic, or Latino and those who self-identified as “other.”

^c^GED: General Education Development.

Overall, there were no significant differences in the demographic items between the intervention and control groups, with the exception of gender: The intervention group was more likely to have male patients than female patients (*P*=.03) Participants who completed the 4-week survey were similar to noncompleters in all demographic variables. As expected, there were some differences in the demographic characteristics of marijuana and opioid users; the opioid users were more likely to be older (*P*<.001) and not in school (*P*<.001).

### Game Rating and Engagement

Intervention participants (n=36) played for an average of 36.6 minutes during the total intervention, of which 35.7 minutes (average) was during inpatient and 0.9 minutes was during outpatient treatment. Participants played for 3.6 days, of which an average of 3.4 days was during inpatient treatment and 0.2 days was during outpatient treatment. Only 3 intervention participants (8.3%) played the game during the outpatient period. For these participants, the average number of outpatient game play days was 2 days, and the average number of game play minutes was 0.9 minutes (Table 2).

Among the intervention group participants who completed the discharge survey (n=32, 88.9%), participants expressed views on their game play preferences: Recovery Ninja was most frequently rated as the most helpful game, followed by Recovery Runner+Goodies. Participants noted that the most helpful mode of game play was avoiding drugs while collecting goodies (eg, Recovery Runner+Goodies), followed by destroying drugs (eg, Recovery Ninja).

The games used the phrase “I’m Clean” to train participants on drug-refusal skills. At 4 weeks of follow-up, among intervention participants who completed the survey (n=23, 63.9%), the majority of participants agreed or strongly agreed that they could imagine using the phase “I’m Clean” in their real lives to refuse drug offers, and the majority stated that when they were not playing the game, the phrase “I’m Clean” rang in their head either a few times a week or daily. Finally, the slight majority (52.2%) stated that they had used the phrase “I’m Clean” to refuse drugs since leaving inpatient treatment.

**Table 2:** Game rating and engagement in the intervention group.

Measure			Value
**Game rating^a^**			
	**Most helpful game, n (%)**		
		Ninja	8 (26.7)
		Ninja+Goodies	3 (10.0)
		Climber	3 (10.0)
		Racer	0 (0.0)
		Racer+Goodies	0 (0.0)
		Runner	3 (10.0)
		Runner+Goodies	7 (23.3)
		I have no preferences, all equally enjoyable	6 (20.0)
	**Mode of game play most helpful, n (%)**		
		Destroy drugs (Ninja, Racer)	12 (40.0)
		Avoid drugs (Runner, Climber)	4 (13.3)
		Avoid drugs and collect goodies (Ninja/Racer/Runner+Goodies)	14 (46.7)
**Game engagement^b^**			
	**Total game play, minutes**		36.6
		Inpatient minutes	35.7
		Outpatient minutes	0.9
	**Total game play, days**		3.6
		Inpatient days	3.4
		Outpatient days	0.2
**Game refusal skills^c^,** **n (%)**		
	Would use “I’m Clean” to refuse drugs (scale: 1-5 points)		3.5 (1.5)
	**Used “I’m Clean” to refuse drugs**	
		Yes	12 (52.2)
		No	11 (47.8)
	**Phrase “I’m Clean” rings in my head**	
		A few times a week or more	13 (56.5)
		Less than once per week	4 (17.4)
		Not at all	6 (26.1)

^a^Measured at discharge (n=32).

^b^Game engagement items are based on computer records of use (n=36).

^c^Measured at 4 weeks postdischarge (n=23).

### Self-Efficacy, Craving, Treatment Rating, and Treatment Use

Overall, cravings declined for both groups from baseline to the 4-week follow up and to the 8-week follow-up, but the differences between groups were not statistically significant (*P*=.45). Self-efficacy fluctuated slightly between baseline, the 4-week follow-up, and the 8-week follow-up but did not change widely between the intervention and control groups (Table 3). These findings do not support the hypothesis that participants who received Recovery Warrior 2.0 reported greater improvements in the predicted mediators of addiction recovery compared to those in usual care.

Measures of treatment rating and treatment use did not reveal differences between groups. Both groups had similar scores on the Counselor Alliance Scale and on their satisfaction with inpatient and outpatient care. Additionally, the utilization of outpatient services did not differ by group at 4 weeks. By 8 weeks, participants in the intervention group reported having attended more outpatient counseling sessions (10.08 vs 4.80), but the difference did not reach significance (*P*=.19; Table 3).

**Table 3:** Effect of game on psychosocial measures of recovery, treatment rating, and treatment use. All values are presented as mean (SD).

Measure	Discharge		At 4-week follow-up		At 8-week follow-up	
	Intervention (n=32)	Control (n=32)	Intervention (n=23)	Control (n=25)	Intervention (n=23)	Control (n=23)
Self-efficacy^a^	6.8 (2.5)	7.6 (3.5)	6.8 (2.4)	8.2 (3.5)	6.8 (1.9)	6.3 (2.5)
Drug craving^a^	5.4 (2.8)	6.9 (3.5)	4.3 (2.2)	5.1 (3.8)	4.9 (2.6)	5.9 (3.7)
Counselor Alliance Scale^a^	32.3 (11.4)	35.5 (10.3)	35.0 (10.6)	35.8 (7.5)	31.5 (10.3)	36.4 (8.9)
Satisfaction with inpatient care^a^	2.4 (1.2)	2.4 (1.1)	N/A^b^	N/A	N/A	N/A
Satisfaction with outpatient care^a^	N/A	N/A	1.8 (0.8)	2.0 (0.7)	2.1 (1.0)	2.1 (0.9)
Total number of outpatient services used	N/A	N/A	2.7 (1.1)	2.7 (1.0)	2.2 (1.6)	1.7 (1.5)
Number of outpatient counseling sessions attended (in past 30 days)	N/A	N/A	10.2 (5.6)	8.4 (6.6)	10.1 (18.7)	4.8 (5.5)

^a^The values presented are scores.

^b^N/A: not applicable.

### Effect of Game on Drug Use

At the 4- and 8-week follow-up periods, there were no significant differences in the rates of either past 7-day and past 30-day abstinences between groups, considering both imputed and complete cases and marijuana and opioid patients together. Analyses were also repeated while controlling for gender, which was not balanced between groups at baseline. Results were similar for gender-adjusted and unadjusted models. The unadjusted models are presented in Table 4.

Although the differences were not significant in the combined marijuana and opioid analysis, the patients were analyzed separately. For analyses with drug use values imputed for the missing values, at 4 weeks after the intervention, 13 of the marijuana patients in the intervention group (72.22%) reported that they did not use drugs in the past 7 days compared with 6 people in the control group (37.50%; *P*=.04). Other results for marijuana patients (past 30 days at 4 weeks and past 7 and 30 days at 8 weeks) were not found to be significant (*P*=.81). No differences were observed for opioid patients.

**Table 4:** Effect of the game on drug use.

Follow-up survey/measure		Drug use values imputed			Complete cases		
		Intervention (N=36), n (%)	Control (N=44), n (%)	Unadjusted risk ratio (95% CI)	Intervention (N=24), n (%)	Control (N=27), n (%)	Unadjusted risk ratio (95% CI)
**At 4-week follow-up**							
	Not used in the past 7 days	22 (61.1)	19 (43.2)	1.4 (0.9-2.2)	22 (91.7)	19 (70.4)	1.3 (1.0-1.7)
	Not used in the past 30 days	18 (50.0)	17 (38.6)	1.3 (0.8-2.1)	18 (75.0)	17 (63.0)	1.2 (0.8-1.7)
**At 8-week follow-up**							
	Not used in the past 7 days	16 (44.4)	19 (43.2)	1.0 (0.6-1.7)	16 (66.7)	19 (76.0)	0.9 (0.6-1.3)
	Not used in the past 30 days	15 (41.7)	16 (36.4)	1.2 (0.7-2.0)	15 (62.5)	16 (64.0)	1.0 (0.6-1.5)

## Discussion

### Principal Findings

This study represents the first randomized trial of a body motion–activated game targeting drug-relapse prevention for patients who were enrolled in an inpatient treatment program and the first trial of a motion-activated video game aimed at the treatment of addiction in youth in any setting. The program was found to be feasible, primarily in the inpatient setting. Participants in the intervention group played for 3.6 days on an average, which was close to the 4 days of game play target set by the study protocol for inpatient care. Those randomized to the game play group mostly agreed that they would use the refusal skills taught by the game, and a near majority reported that they used those skills 4 weeks after discharge. There was a trend for those in the intervention group to report attending more outpatient counseling sessions than the control group, but the differences were not significant. There was a trend for an effect of the game on past 7- and 30-day drug use at 4 weeks postdischarge, with a significant benefit for a subgroup of participants who were in treatment for marijuana use disorder. No evidence was found that the game worked by differentially improving self-efficacy for drug refusal or reducing cravings.

Overall, the dose of game play was small, limiting the potential for demonstration of effect. Contrary to the intended protocol, most intervention participants never received any game play after discharge from inpatient treatment. Thus, as designed, this study could not address the question of the effects of continued exposure to game play in the outpatient setting. Difficulties encountered for outpatient treatment were largely due to characteristics of the trial. Only about half of the intervention participants came to the MMTC for outpatient care. For the few who did come, game play could only be offered in the outpatient setting on an individual basis, as there were too few trial participants at any given time to form a group. Participants expressed that they did not want to leave their outpatient group counseling sessions for individual game play and therefore declined to play in this setting. Future tests of the game may benefit from careful consideration of group dynamics, and where possible, deliver the game in a group, social format rather than in an individual game format. Furthermore, if patients are unlikely to get access to the game in their outpatient treatment setting, additional opportunities should be developed for game play in other settings, perhaps using home-based play on a computer or smartphone. This may have the potential to make the effects of the game last longer and should be investigated further.

We found some effect of the game for marijuana participants but not opioid participants; this may indicate that the game is more promising for the former subgroup. It is possible that these patients are younger, with lower addiction severity and chronicity, and more likely to respond to a behavioral intervention. A game may also be more consistent with younger patients’ preferences for less “serious” and more experiential treatments. It may be that higher doses of game play are needed for more entrenched physiological addiction such as that for opiates.

Although originally hypothesized as mediators of the effect of the game, the game play did not appear to increase the levels of self-efficacy for drug refusal, as self-efficacy remained constant. It is possible that the game does not operate as hypothesized through drug refusal self-efficacy. It also appears that the game does not differentially decrease cravings. Other mechanisms such as repetition priming can be explored as mechanisms in future studies of the game.

### Strengths

Strengths of this study include that it was the first randomized study of a motion-activated video game aimed at the treatment of addiction in youth. The game was built around an affordable off-the-shelf motion-sensing peripheral—Microsoft Kinect—widely used by youth, which is most famous for its use with Microsoft’s popular Xbox video game platform. The potential for dissemination is high, with the possibility for play not only in treatment centers, but also at home.

### Limitations

The study experienced significant loss to follow-up, as about 40% of participants were not available for the 4-week and the 8-week follow-up interviews. Although this is a high level of attrition, this level is not unusual for youth attending drug-treatment facilities, as participants following discharge are at high risk for dropout, relapse, incarceration, or readmission to inpatient treatment. Additionally, marijuana and opioid patients were found to have different demographic characteristics and possibly different responses to the game. In addition, treatment adherence in the intervention group was low in the postdischarge period, and few participants experienced game play after leaving the inpatient setting. It should also be noted that while current video game use was captured in this study, the use of Microsoft Kinect specifically was not captured and may have implications for the dose and fidelity of the intervention. Another limitation is that the intervention group was not balanced with the control group for gender, as there were fewer female patients in the intervention group than in the control group, although we did not find different effects of the game by gender. Finally, the drug use outcome measures in this study relied on self-report only and because of social desirability, they may represent undercounts of relapse rates. Future studies should use drug testing to verify abstinence.

### Conclusions

This pilot study provides encouraging proof-of-concept results to show that an early prototype of the Recovery Warrior game is feasible and acceptable in inpatient treatment settings and produces some encouraging outcomes. Future larger studies with a more refined version of the game are warranted to test its implementation in outpatient treatment settings, its overall efficacy, and how to best adapt it to different drug-using subgroups.

## Appendix

Multimedia Appendix 1 Details and screenshots of the games.

Multimedia Appendix 2 CONSORT-EHEALTH checklist (V 1.6.1).

## Abbreviations

GED: General Education Development
MMTC: Mountain Manor Treatment Center
SUD: substance use disorder
