# Supplementary material for: A Motion-Activated Video Game for Prevention of Substance Use Disorder Relapse in Youth: Pilot Randomized Controlled Trial
Source: JMIR Serious Games. 2019 May 23;7(2):e11716. doi: 10.2196/11716 (PMC6552452; doi:10.2196/11716)
Supplement: Multimedia Appendix 1 [file games_v7i2e11716_app1.pdf]

Appendix 1.

i. **Recovery Ninja** (destroy; destroy + discern): The *Recovery Warrior* avatar must destroy drugs and drug paraphernalia flying toward him by using chopping, punching, and hitting gestures in order to win the game. In destroy mode, if the player fails to destroy all the drugs that appear and allows drugs to get past him, the player's avatar loses Health, shown in a red Health bar. The more Health the avatar loses, the more the avatar's body motions slow down, making it more challenging to continue the mission; this reflects the reality in which drugs negatively impact physical and cognitive abilities. If the avatar loses all Health, the game ends. The player must repeatedly vocalize "I'm Clean" to regain Health to continue playing the game. The player wins by not losing all Health for 60 seconds. In discern mode, the flying drugs are mixed with flying "goodies" objects (i.e. graduation caps, car keys, engagement rings, footballs, etc.), which represent the activities and accomplishments a player can enjoy and achieve as an alternative to drugs. This will require the player to pick up goodies while avoiding all drugs. This allows the player to practice discerning between drugs and "goodies."

Figure 1. Recovery Ninja in Destroy Mode

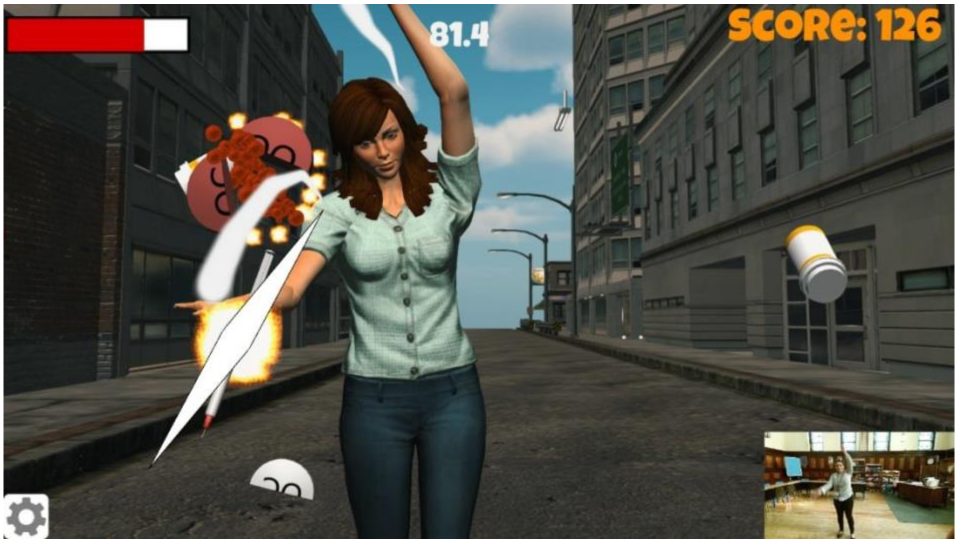

Note: The 1:1 correspondence of player motion (lower right) to avatar

ii. **Recovery Runner** (avoid; avoid + discern): The *Recovery Warrior* avatar runs through a dark three-dimensional city, which progressively brightens as the player succeeds in staying away from drugs. In avoid mode, drugs and drug paraphernalia appear as obstacles along the path ahead. Instead of destroying drugs (as in Recovery Ninja and Recovery Racer), the avatar must avoid the drugs by physically ducking, dodging, and jumping. If the player's avatar bumps into drugs, the avatar loses Health, shown in a red Health bar. If the avatar loses all Health, the game ends. A special feature in this game allows the player to extend their hands forward in a "stop" gesture to slow down to make

maneuvering easier, but only when their “Speed” bar is charged up (players charge the bar by avoiding drugs). At higher difficulty settings, players can speed up by extending their arms to the sides, like wings. This makes skillful player able to gain higher scores. The player must repeatedly vocalize “I’m Clean” to regain health to continue playing the game. In discern mode, the drugs along the path ahead are mixed with trails of “goodies,” which the player’s avatar collects by running through them. The player must skillfully evade the drugs along the path while heading toward the goodies to collect them. This allows the player to practice discriminating between drugs and “goodies.”

Figure 2. Recovery Runner in Avoid + Discern Mode

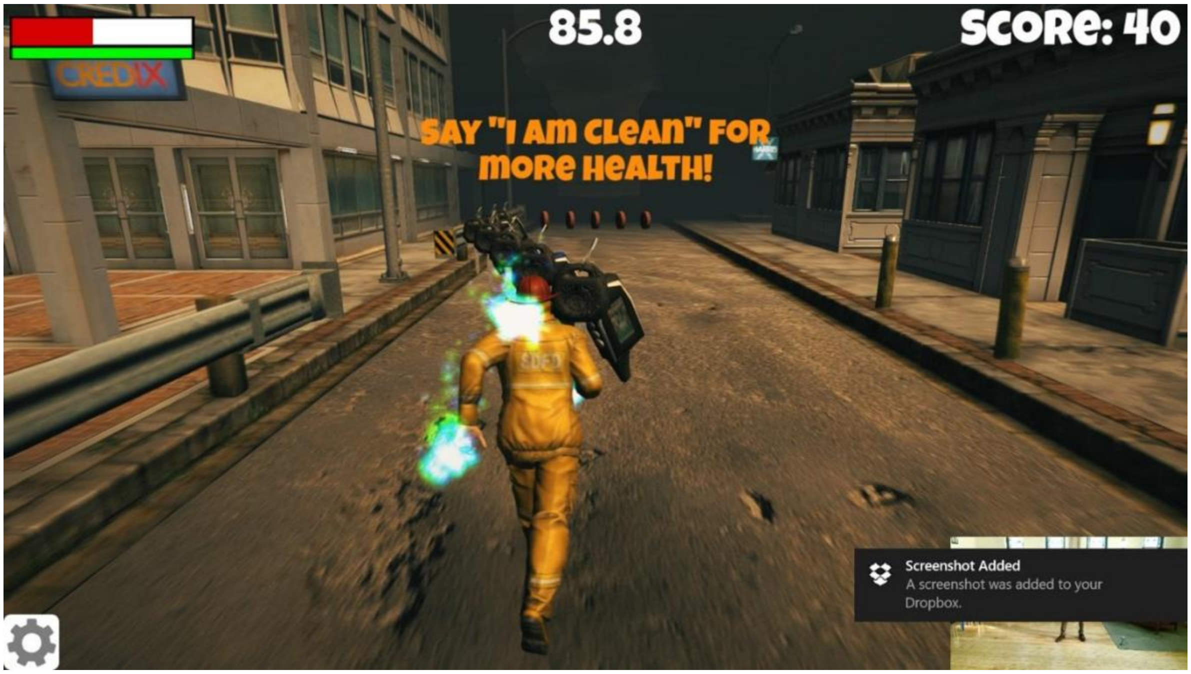

Note: The car keys in foreground, drugs in background

iii. **Recovery Climber** (avoid + transform): The *Recovery Warrior* avatar must climb a building bristling with drugs and drug paraphernalia towards safety and friends on the roof by following a pathway of safe, glowing handholds. Players climb the building by using upward pulling, grasping, downward pulling, and releasing motions. They can climb down by using the same motions in reverse. They can also traverse the building sideways. The player must grasp the glowing handholds and avoid using the surrounding drugs as a handhold. Climbing by grasping drugs instead of safe handholds reduces the health of the player’s avatar, shown in a red Health bar. If the avatar runs out of Health, the avatar falls. The player also has a blue Energy bar, which decreases as the player hangs on a handhold. When the player grabs a new handhold, the blue energy bar fills again. If the blue Energy bar runs out, the avatar falls. The energy bar does not reduce in Casual difficulty mode, so even patients with some motion deficits can play. The player can enter doors marked “Rehab” to gain a power-up which lets them

eliminate the drugs in their path, transforming them to safe handholds by vocalizing “I’m Clean!”

Figure 3. Recovery Climber in Transform Mode

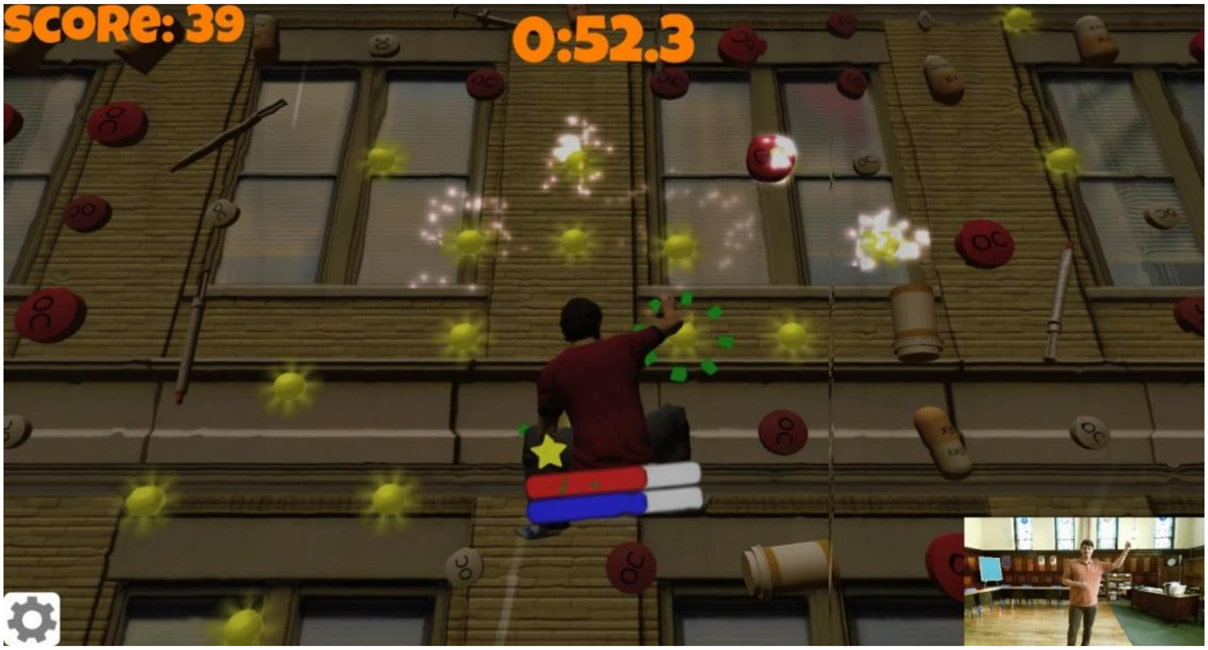

iv. **Recovery Racer** (destroy; discern): The *Recovery Warrior* avatar uses a motorcycle to drive through a city where the roads are littered with drugs and drug paraphernalia. She can destroy the drugs along the speeding course by running over them. Hand and arm motions are required to steer the motorcycle and a kick motion speeds it up. The player must repeatedly vocalize “I’m Clean” to gain a short-term power-up which lets her destroy drugs by riding near the drugs without having to precisely impact them. (Note: For players who don’t like to lose, Recovery Racer does not have a Health setting or a loss condition, so all players can complete the 60 second race on all difficulty modes without fear of losing.) In discern mode, “goodie” items appear along the road and the player must drive over them to collect them. These “goodies” (i.e. graduation caps, car keys, footballs, etc.), represent the accomplishments a player can achieve as an alternative to drugs. This will require the player to pick up goodies while avoiding all drugs. This allows the player to practice discriminating between drugs and “goodies.”

Figure 4. Recovery Racer in Destroy Mode

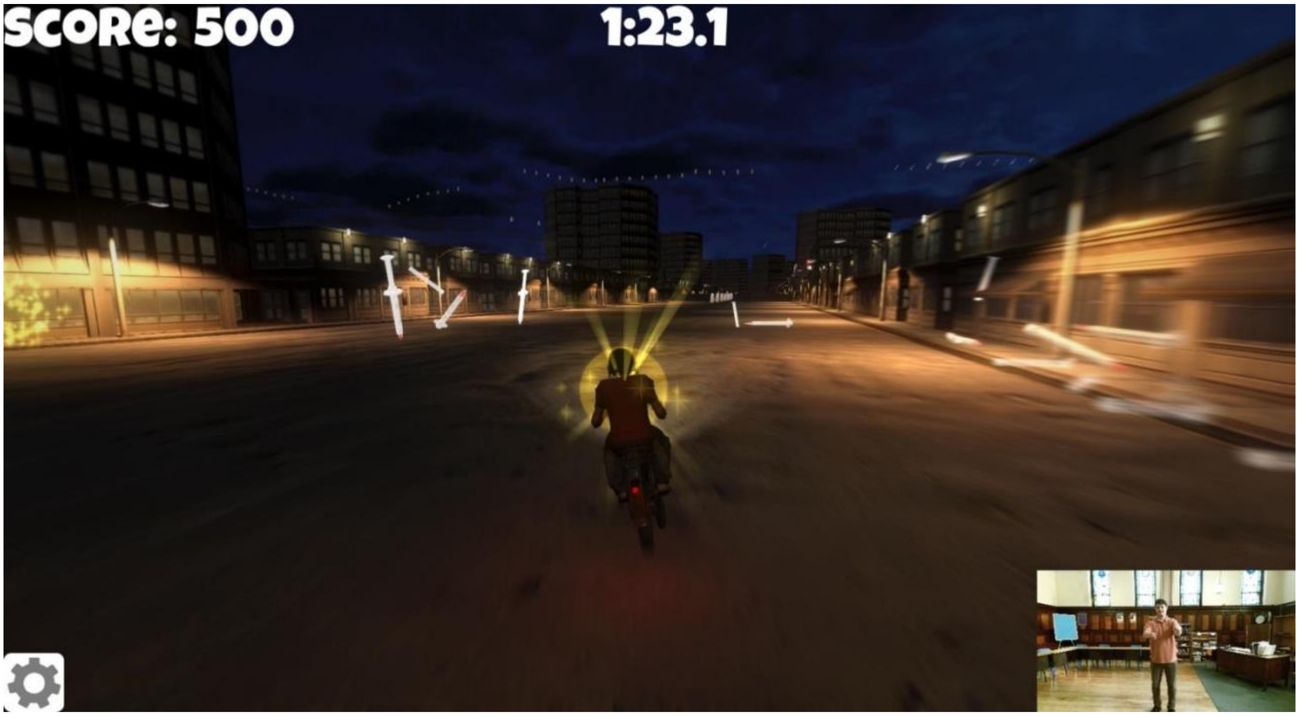

Note: The glow indicates that the player said "I'm Clean" to expand their drug busting power
